# Dietary management and major clinical events in patients with longchain fatty acid oxidation disorders enrolled in a phase 2 triheptanoin study

**DOI:** 10.1016/j.clnesp.2020.11.018

**Published:** 2020-12-25

**Authors:** Jerry Vockley, Nicola Longo, Megan Madden, Lauren Dwyer, Yunming Mu, Chao-Yin Chen, Jason Cataldo

**Affiliations:** a University of Pittsburgh School of Medicine, UPMC Children’s Hospital of Pittsburgh, 4401 Penn Avenue, Pittsburgh, PA 15238, USA; b Department of Pediatrics School of Medicine, University of Utah, 30 N 1900 E, Salt Lake City, UT, USA; c Ultragenyx Pharmaceutical Inc., 60 Leveroni Ct, Novato, CA, USA

**Keywords:** Triheptanoin, Medium-chain triglyceride, Acute metabolic crises, Rhabdomyolysis, Hypoglycemia, Cardiomyopathy

## Abstract

**Background & aims:**

Long-chain fatty acid oxidation disorders (LC-FAOD) are rare, life-threatening, autosomal recessive disorders that lead to energy depletion and major clinical events (MCEs), such as acute metabolic crises of hypoglycemia, cardiomyopathy, and rhabdomyolysis. The aim of this study was to report a post hoc analysis of diet diary data from the phase 2 UX007-CL201 study (NCT01886378).

**Methods:**

In the single-arm, open-label, phase 2 UX007-CL201 study, the safety and efficacy of 78 weeks of treatment with triheptanoin, an odd-carbon, medium-chain triglyceride consisting of three 7-carbon fatty acids on a glycerol backbone, was investigated in subjects with LC-FAOD versus a retrospective 78-week period when subjects were optimally managed under published dietary guidelines. Subject dietary reports were collected to analyze the relationship between diet, triheptanoin treatment, and MCEs. Referring metabolic physicians completed a survey on patient management and clinical outcomes before and after initiation of triheptanoin. Before initiating triheptanoin, subjects received a mean daily caloric intake (DCI) of 17.4% from medium-chain triglycerides (MCT). During the study, subjects received a mean of 27.5% DCI from triheptanoin. Protein (13.7% vs 14.5% DCI), long-chain fat (13.1% vs 10.5% DCI), and carbohydrate (55.3% vs 47.1% DCI) intake were consistent between the pre-triheptanoin and triheptanoin treatment periods, respectively.

**Results:**

Following 78 weeks of treatment, mean annualized MCE rate decreased by 48.1% (p = 0.021) and mean annualized MCE event-day rate decreased by 50.3% (p = 0.028). A weak association existed between improvement in annualized MCE rate and change in percent DCI from MCT (Spearman rank correlation: *r* = −0.38; 95% CI: −0.675, 0.016). However, there was large variability in the association and no specific pattern of change for larger or smaller changes in dose. Seventy-two percent of physicians reported that triheptanoin had a clinically meaningful benefit on medical management of their patients.

**Conclusions:**

Treatment with triheptanoin at the protocol-specified dose decreased the rate of MCEs in patients with LC-FAOD independently from other dietary changes between the pre-triheptanoin and triheptanoin treatment periods.

*Trial registration:* ClinicalTrials.gov identifier: NCT01886378.

## 1. Introduction

Long-chain fatty acid oxidation disorders (LC-FAODs) are rare, serious, and life-threatening autosomal recessive disorders caused by defects in the metabolic pathway that converts fatty acids into energy. These defects can lead to depleted energy sources in patients, resulting in severe acute metabolic crises, such as rhabdomyolysis, hypoglycemia, and cardiomyopathy, which may lead to hospitalizations or early death [1–4]. LC-FAODs include inherited defects in one of six different genes coding for carnitine palmitoyl transferase 1a (CPT-Ia; *CPT1A*), carnitine palmitoyl transferase 2 (CPT-II; *CPT2*), carnitine/acylcarnitine translocase (CACT; *SLC25A25*), very long-chain acyl-CoA dehydrogenase (VLCAD; *ACADVL*), long-chain 3 hydroxyacyl-CoA dehydrogenase (LCHAD; *HADHA*), and mitochondrial trifunctional protein (TFP; a heterodimer encoded by *HADHA* and *HADHB*).

Published disease management involves strict avoidance of fasting and a low-fat/high-carbohydrate diet, with medium-chain triglycerides (MCT) and carnitine supplementation [5]. These treatments have not been formally studied in controlled clinical trials and represent approaches developed by physicians using case experience. Diet modification and fasting avoidance can benefit some patients with LC-FAOD; however, hypoglycemic events, exercise intolerance, muscle weakness, rhabdomyolysis, and cardiomyopathy still commonly occur [2,3,6,7]. These events remain associated with a high mortality rate, highlighting a serious unmet medical need [1,2].

Triheptanoin (Dojolvi) is an odd-carbon, medium-chain triglyceride consisting of three 7-carbon fatty acids on a glycerol backbone, intended as an energy substrate replacement therapy for the treatment of LC-FAOD. Triheptanoin is approved in the United States for the treatment of pediatric and adult patients with molecularly confirmed LC-FAOD. Triheptanoin is metabolized to seven-carbon fatty acids that can diffuse across the mitochondrial membrane and are further metabolized into propionyl-CoA and acetyl-CoA by mediumand short-chain fatty acid oxidation enzymes, bypassing the enzymes that are deficient in LC-FAOD. The production of propionyl-CoA is responsible for the anaplerotic characteristics of triheptanoin [8,9], which are believed to be critical for the tricarboxylic acid cycle, gluconeogenesis, and restoring the energy deficiency in LC-FAOD.

The single-arm phase 2 UX007-CL201 study (NCT01886378) was conducted in 29 pediatric and adult subjects with LC-FAOD to prospectively evaluate the safety and efficacy of 78 weeks of triheptanoin treatment compared with a retrospective 78-week pretriheptanoin period [10,11]. Compared with the pre-triheptanoin period, 78 weeks of triheptanoin treatment reduced the rate of major clinical events (MCEs; e.g., hypoglycemia, rhabdomyolysis, cardiomyopathy), maintained improvements in walking exercise tolerance, and increased health-related quality of life [10]. Here, we present additional analysis from this phase 2 study of dietary data during the 4-week run-in period, baseline, and triheptanoin treatment periods, as well as analysis of referring physician questionnaires.

## 2. Subjects and methods

### 2.1. Study description

This study enrolled 29 subjects aged ≥6 months with symptomatic LC-FAOD. The subjects had confirmed diagnoses of CPT-II, VLCAD, LCHAD, or TFP deficiency (Supplementary Table 1). The study was conducted in compliance with the Declaration of Helsinki, relevant institutional review board practices, and the International Conference on Harmonisation Good Clinical Practice guidelines. Informed consent was obtained for experimentation with human subjects. Further details of this study have previously been published [10,11].

Study guidelines were developed based on input from expert metabolic physicians and dietitian consultants to support a safe transition from MCT to triheptanoin and minimize variability in macronutrient breakdown. As a part of this transition, subjects increased their daily caloric intake (DCI) from medium-chain fat (i.e., triheptanoin) compared with prestudy MCT or no MCT. The study drug administration guidelines recommended that subjects follow a diet consisting of 25–35% DCI from triheptanoin, ~15% DCI from protein, an appropriate percentage of DCI from long-chain fatty acids to meet essential fatty acid requirements, and the balance of energy in the form of carbohydrates.

### 2.2. Outcome measures

The key study endpoints in the present analysis include macronutrient breakdown before and after triheptanoin initiation, frequency and duration (expressed as event-days) of MCEs, exercise tolerance, health-related quality of life, and safety.

The macronutrient breakdown (protein, carbohydrate, total fat, medium chain fat, and long chain fat) was measured as the percentage of total DCI before and during triheptanoin initiation. Pretriheptanoin levels were calculated from the 3-day diet diary data obtained during the run-in period and baseline visit. During-triheptanoin levels were calculated from data obtained during the triheptanoin treatment period at scheduled study visits (weeks 12, 24, 48, and 78).

Reductions in rates and study-days of MCEs, and changes in exercise tolerance and quality of life measures were collected from surveys on patient management and clinical outcomes completed by referring metabolic physicians both before and after initiation of triheptanoin.

### 2.3. Dietary assessments

Three-day diet records were collected during three study periods: 1) during a 4-week run-in period in which subjects maintained current therapy (including medium even-chain triglycerides); 2) at baseline before subjects initiated triheptanoin; and 3) during the triheptanoin treatment period at scheduled study visits (weeks 12, 24, 48, and 78). The diet diaries were analyzed using metabolic software (MetabolicPro [United States] and Microdiet [United Kingdom]) to calculate the subject’s total calories and macronutrient distribution. Each subject’s metabolic needs were calculated by a metabolic dietitian, who took into account the patient’s individual dietary restrictions, age, weight, and clinical condition. During the study, each subject’s diet remained relatively isocaloric, allowing for consistency in percentage of fat, protein, and carbohydrates. This method of dietary analysis is routinely used in clinical practice [2] and was adopted to minimize the impact of introducing triheptanoin as a source of medium-chain fat during the study that may have confounded treatment effects. Formal dietary assessments were not recorded in these diaries during the entire 78-week retrospective period, as this was not required per protocol. Regular dietary management per guidelines was performed during this retrospective period as part of routine medical care for LC-FAOD.

### 2.4. Physician questionnaire

Referring metabolic physicians who managed subjects prior to study enrollment were invited to complete a survey on the medical management of their subjects prior to initiation of triheptanoin in this phase 2 study (Supplementary Appendix). The focus of this questionnaire was to obtain information related to the subject’s LCFAOD, the number of clinic visits, MCT use, dietary compliance, and dietary optimization prior to enrollment in this phase 2 study. Additionally, physicians who continued overseeing subjects enrolled in the study were asked their opinion on the effect of triheptanoin on overall patient management and clinical condition.

### 2.5. Statistical analysis

The annualized event rate (events/year) and annualized eventday rate (days/year) were calculated for the 78 weeks before triheptanoin initiation and during triheptanoin treatment. The comparison of annualized event and event-day rates between pretriheptanoin and during-triheptanoin periods was analyzed using the paired *t*-test and the corresponding p-values were calculated.

The relationship between mean annualized MCE rate and change in proportion of DCI from medium chain fat was examined using a Spearman’s rank correlation.

## 3. Results

### 3.1. Published dietary guidelines for LC-FAOD

A key objective of this study was to compare triheptanoin at 25–35% DCI with previously established dietary management. Patients with LC-FAOD conditions are managed by dietitians with expertise in inborn errors of metabolism. Energy needs and macronutrient distribution for each patient are estimated based on the individual’s gender, age, weight, activity level, and clinical status. After estimating adequate calories (kcals), appropriate levels of protein are calculated; for infants and children, growth and development must be considered. The amount of protein prescribed typically provides ~15% DCI, although a higher percentage has been reported to be beneficial in LC-FAOD [7]. The amount of total, medium-chain, and long-chain fats are then planned in accordance with published guidelines (Table 1).

### 3.2. Dietary analysis: macronutrient breakdown and DCI

**Fig. 1.**
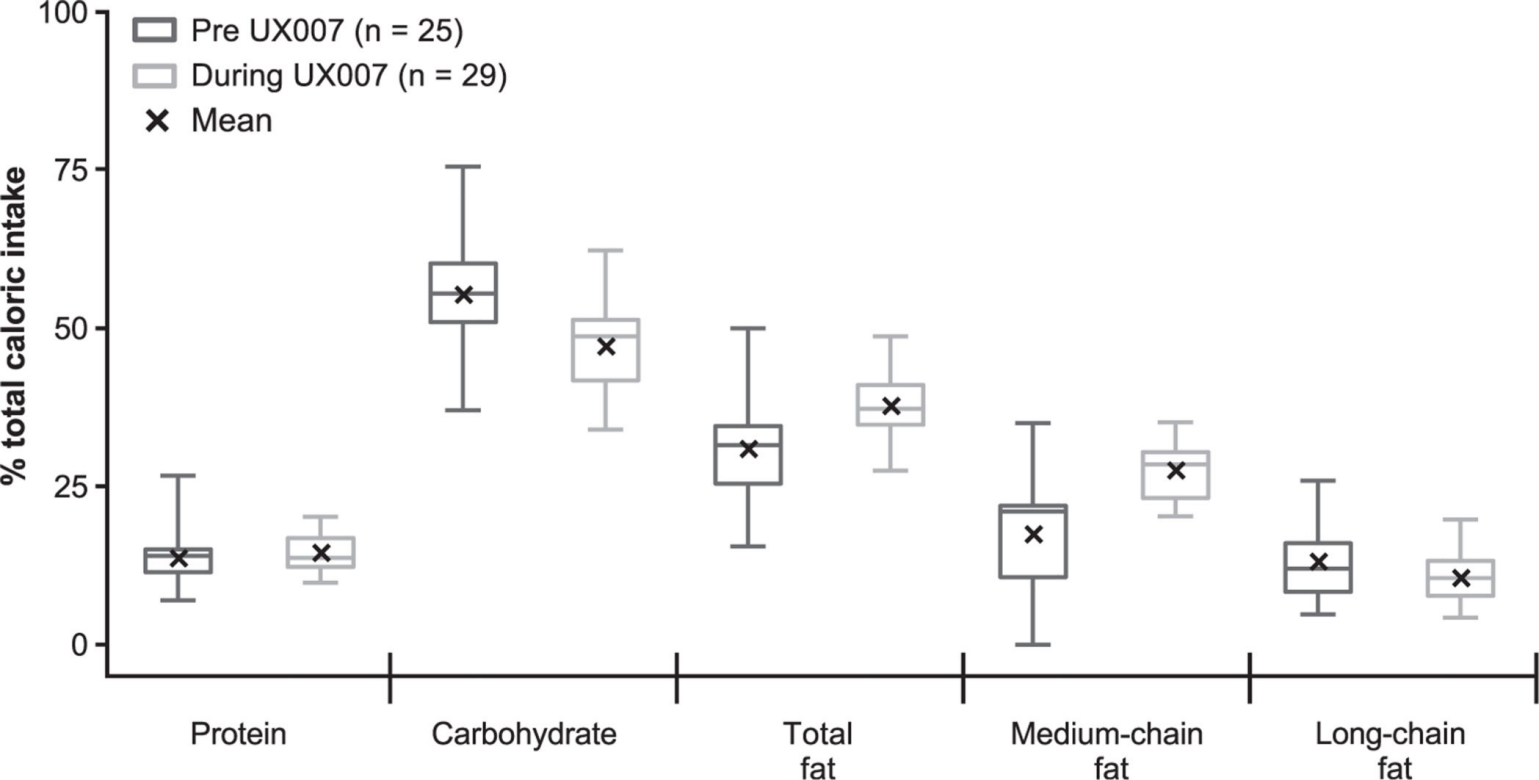
Total daily macronutrient distribution before and during triheptanoin therapy. Box and whisker plot shows the minimum, lower quartile, median, upper quartile, and maximum values. The mean is represented with an “x.” Pre-triheptanoin treatment period values are based on available data during the run-in and baseline visits. Triheptanoin treatment period values are based on available data during the week 12, 24, 48, and 78 visits.

As required by the study protocol, subjects increased DCI from medium-chain fat (i.e., triheptanoin) compared with prestudy MCT intake or no MCT (Table 2). The 3-day diet diary data captured during the run-in period and baseline visit reflect subjects’ calorie intake and macronutrient distribution prior to study enrollment and were consistent with published dietary guidelines (Fig. 1; Tables 1 and 2).

The percentage of DCI for protein (13.7% vs. 14.5%) and longchain fat (13.1% vs. 10.5%) was similar between the pre-triheptanoin and triheptanoin treatment periods (Table 2). On average, subjects had an ~10% increase in DCI from medium-chain fat (17.4% vs. 27.5%), and an ~8% decrease in DCI from carbohydrates (55.3% vs. 47.1%). Total DCI increased from ~62 to 72 kcal/kg, driven by energy requirements for growth and activity.

### 3.3. Impact of dietary change on MCEs

**Fig. 2.**
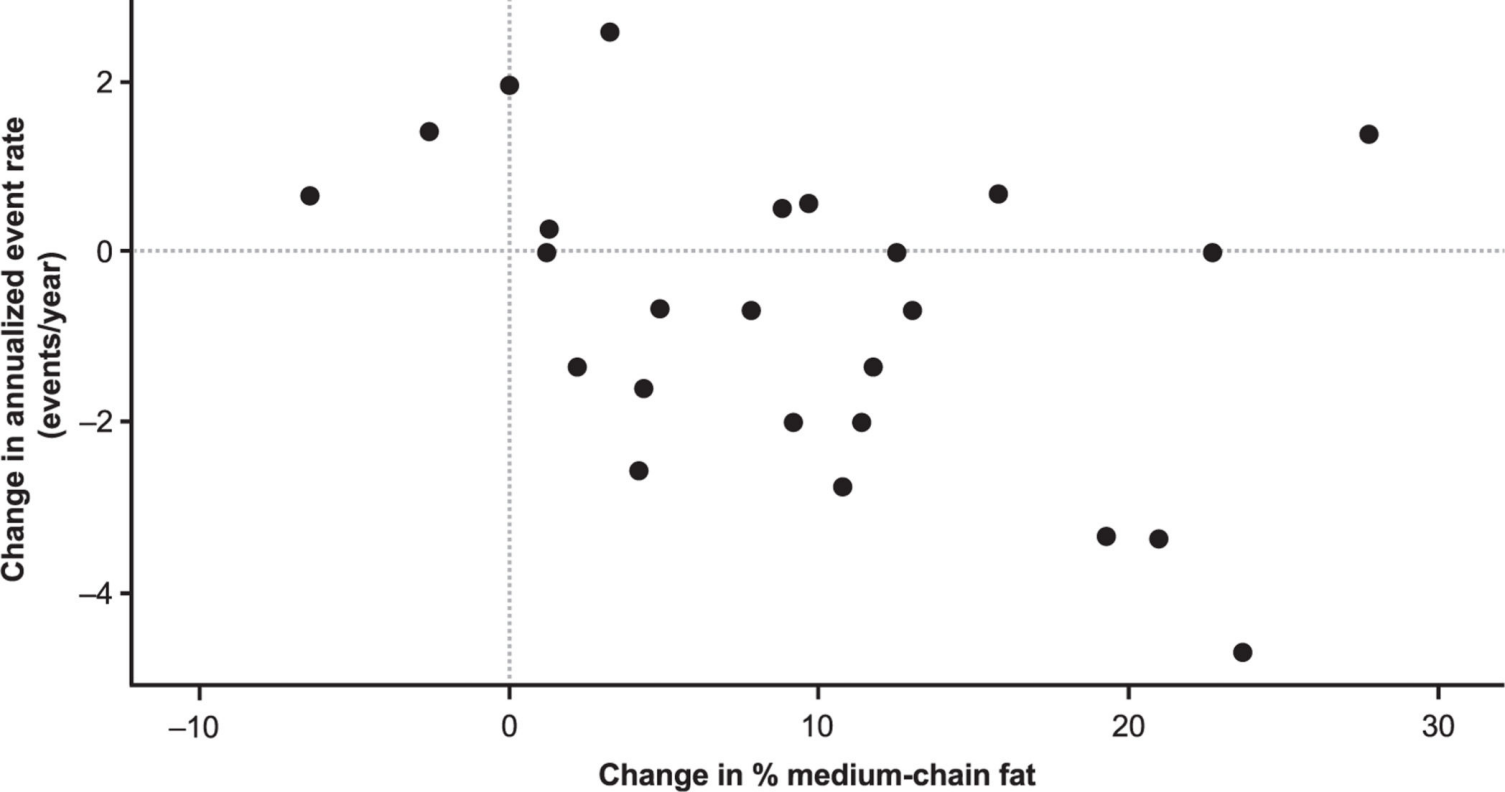
Change in major clinical events and medium-chain fat intake. Spearman rank correlation: *r* = −0.38 (95% CI: −0.675, 0.016).

Subjects who crossed over to triheptanoin at a target therapeutic dose range of 25–35% DCI experienced a clinically meaningful improvement in mean annualized MCE rate from 1.69 to 0.88 events/year (48.1% reduction; p 0.0208) and mean annualized MCE event-day rate from 5.96 to 2.96 days/year (50.3% reduction; p 0.0284; Supplementary Table 2) [10]. A weak association was observed between changes in percent DCI from MCT to triheptanoin and MCE improvement that did not reach statistical significance (Spearman rank correlation: *r =*−0.38; 95% CI: −0.675, 0.016; Fig. 2). The increase in DCI from even-chain MCT to triheptanoin reflects the protocol-specified regimen. Furthermore, when evaluating subjects who had a reduction in events while taking triheptanoin, the change in dose ranged widely from a few percentage points to a 25% increase in medium-chain fat.

### 3.4. Analysis of physician questionnaires

Responses to the questionnaire regarding the 78-week retrospective period were received from 17 physicians at 16 different metabolic centers for 25 (86%) of 29 subjects enrolled in this study. Questionnaire responses for four subjects were not obtained because two of the referring physicians had retired at the time of the request and two referring physicians did not respond.

Based on completed physician surveys (n 25), almost all subjects were managed by a metabolic physician (96%) and all were managed by a metabolic dietitian (100%) during the retrospective 78-week pretreatment period (Table 2). Clinical assessments occurred frequently during this time, with most subjects (76%) assessed at four or more separate clinic visits. One subject did not have any oversight in the 78 weeks prior to enrollment because the subject chose not to be under physician care and was not on MCT prior to the study. Most subjects (92%) were on MCT during the retrospective 78 weeks, and the overall pretreatment average MCT dose ranged from 0 to 37% DCI.

In the view of the referring physicians, 84% of subjects were compliant with dietary management prior to study initiation, and 80% thought treatment was medically optimized in the 78 weeks prior to initiation of triheptanoin (Table 3). Five subjects were identified as not being medically optimized via diet, including two subjects who were not taking MCT due to subject choice and three subjects who continued to have clinical events despite dietary management. Repeated hospitalizations were often cited as a measure of nonoptimization, but lack of dietary compliance or management oversight were not provided as explanations for any subject not being medically optimized.

The referring physicians also provided perspective on several clinical outcomes during the 78-week triheptanoin treatment period, including the reduction in the rate and event-days of MCEs, exercise tolerance, and quality of life. Specifically, they were asked whether the benefit observed with triheptanoin at its target dose of 25−35% DCI compared with prior management was clinically meaningful. The majority of physicians (18/25; 72%) responded that the effect of triheptanoin was clinically meaningful, had an impact on medical management, and demonstrated an improvement in subjects (Table 3). For the remaining 28% (7/25) of subjects, the physician did not respond (2/25; 8%), did not see improvement while on study (2/25; 8%), indicated that they both did and did not see improvement while on study (1/25; 4%), or was not the treating physician for the patient following study enrollment (2/25; 8%).

## 4. Discussion

Triheptanoin decreased the rate of MCEs in patients with LCFAOD compared with established pretreatment management in the phase 2 study [10]. Although the subjects had an ~10% increase in DCI from medium-chain fat after transitioning to triheptanoin, this increase was expected as triheptanoin was dosed, per protocol, at a higher percentage than clinical guidelines recommend for MCT [2,5,12,13]. Macronutrient breakdown was consistent during the combined 4-week run-in and baseline periods and remained stable during the triheptanoin treatment period. The dietary analysis supports that the change in treatment regimen from previously established pretreatment management to triheptanoin at a dose range of 25–35% DCI resulted in a significant improvement in the rate and event-days of MCEs. Reductions in MCEs were observed across the spectrum of change in medium-chain fat intake, indicating that this may not be the primary cause of improvement following the transition to triheptanoin.

The metabolic physicians and dietitians who managed subjects during the retrospective 78-week period before study enrollment indicated that most subjects were medically optimized via diet and demonstrated good compliance with their individualized management. The referring metabolic physicians who continued to manage their patients during the study believed that triheptanoin dosed at 25–35% DCI demonstrated a clinically meaningful improvement and positive effect on their patients’ outcomes compared with their prior dietary treatment regimens.

The results presented in the current report suggest that the improvement in outcomes was unlikely caused by concurrent changes in diet or clinical care, but rather caused by the unique properties of triheptanoin benefitting subjects with LC-FAOD.

### Data sharing

Due to the rarity of LC-FAOD and the small number of subjects in this trial, individual patient data will not be shared in order to safeguard patient privacy, consistent with the data sharing policy listed on Ultragenyx.com. This trial was registered at www.clinicaltrials.gov as NCT01886378.

## Supplementary Material

Supplement 1

Supplement 2

## Figures and Tables

**Table 1:** Published dietary fat intake guidelines for patients with LC-FAOD.

Publication	Total fat	Medium-chain triglycerides		Long-chain triglycerides	
	% of kcals	% of kcals	g/kg/day	% of kcals	g/kg/day
Rohr et al. (2008)	35	15–25	2–3	10–20	NA
Spiekerkoetter et al. (2009a)	25–30	20–25	NA	5–10	NA
Spiekerkoetter et al. (2009b)	NA	NA	0.7–3.3 (VLCAD)	NA	0.8–2.1 (VLCAD)
			0.5–2.6 (LCHAD)		0.6–2.3 (LCHAD)
			1.8–4 (TFP)		0.5 (CPT-II)
Rohr(2015)	NA	10–30	2–3(≤1 year)	8–10	NA
			1–1.25 (>1 year)		

CPT-II = carnitine palmitoyl transferase 2; LCHAD = long-chain 3 hydroxyacyl-CoA dehydrogenase; LC-FAOD = long-chain fatty acid oxidation disorders; NA = not available; TFP = trifunctional protein; VLCAD = very long-chain acyl-CoA dehydrogenase.

**Table 2:** Summary of 3-day diet diary for the pre-triheptanoin and triheptanoin treatment periods.

Statistics	Total caloric intake (kcal/kg/day)	Protein (% of total caloric intake)	Carbohydrate (% of total caloric intake)	Total fat (% of total caloric intake)	Medium-chain fat (% of total caloric intake)	Long-chain fat (% of total caloric intake)
Pre-triheptanoin treatment period average^a^						
n	29	29	29	29	29	29
Mean (SD)	61.5 (30.0)	13.7 (4.3)	55.3 (9.0)	30.9 (8.2)	17.4 (8.9)	13.1 (6.2)
Median	60.7	14.0	55.5	31.5	21.0	12.0
Q1, Q3	32.0, 83.9	11.0, 15.5	50.5, 60.7	25.0, 35.0	10.2, 22.4	7.9, 16.5
Min, max	20.8, 122.5	7.0, 26.7	37.0, 75.5	15.5, 50.0	0.0, 35.0	4.8, 25.9
Pre-triheptanoin treatment period average (subjects with both preand postbaseline dietary data)^b^						
n	25	25	25	25	25	25
Mean (SD)	63.9 (30.8)	14.0 (4.4)	54.0 (8.7)	31.7 (8.3)	18.0 (8.8)	13.3 (6.2)
Median	67.2	14.0	54.6	32.0	21.2	12.0
Q1, Q3	32.5, 84.5	11.0, 16.0	49.0, 58.1	26.0, 35.5	10.2, 22.4	8.4, 16.5
Min, max	20.8, 122.5	7.0, 26.7	37.0, 75.5	15.5, 50.0	0.0, 35.0	4.8, 25.9
Triheptanoin treatment period average^c^						
n	25	25	25	25	25	25
Mean (SD)	71.9 (38.1)	14.5 (3.3)	47.1 (7.4)	37.7 (5.7)	27.5 (4.6)	10.5 (3.8)
Median	68.3	13.7	48.7	37.3	28.5	10.5
Q1, Q3	35.9, 100.7	11.8, 17.3	41.3, 51.8	34.3, 41.5	22.7, 30.9	7.3, 13.7
Min, max	17.8, 151.7	9.8, 20.2	34.0, 62.3	27.5, 48.7	20.3, 35.1	4.3, 19.8

max = maximum; min = minimum; Q = quartile.

a Pre-triheptanoin treatment period values are based on average value of available data during the run-in and baseline visits.

b Pre-triheptanoin treatment period values are based on average value of available data during the run-in and baseline visits using only subjects with both preand postbaseline dietary information.

c Triheptanoin treatment period average values are based on average value of available data across the week 12, 24, 48, and 78 visits.

**Table 3:** Physician responses to questions about pretreatment diet and response to triheptanoin therapy.

Survey question	n (%)^a^	Comments
Managed by a metabolic physician	24 (96)	One (4%) subject was not under physician care and was not taking MCT during this period
Managed by a metabolic dietitian	25 (100)	
Number of interactions with subject		
1–3 interactions	4 (16%)	One (4%) subject had no data available; one (4%) subject was not under physician care
4–6 interactions	12 (48%)	
7–9 interactions	2 (8%)	
≥10 interactions	5 (20%)	
On MCT treatment before enrollment	23 (92%)	Two (8%) subjects not taking MCT due to patient choice
Compliant with the pre-treatment diet	21 (84%)	
Medically optimized via diet in the 18 months prior to enrollment	20 (80%)	
Believe improvement observed in the study was due to triheptanoin treatment	18 (72%)	Two physicians did not respond; two physicians responded “no”; two physicians responded “NA”; one physician responded both “yes” and “no”

Responses to the questionnaire regarding the 78-week retrospective period were received from 17 physicians at 16 different metabolic centers for 25 (86%) of 29 subjects enrolled in this study.

MCT = medium-chain triglycerides; NA = not applicable.

a n = 25: questionnaire responses for four of 29 subjects were not obtained; two of the referring physicians had retired at the time of the request and two referring physicians did not respond.

## Abbreviations:

CPT-II: carnitine palmitoyl transferase 2
DCI: daily caloric intake
LC-FAOD: long-chain fatty acid oxidation disorder
LCHAD: long-chain 3 hydroxyacyl-CoA dehydrogenase
MCE: major clinical event
MCT: medium-chain triglyceride
TFP: trifunctional protein
VLCAD: very long-chain acyl-CoA dehydrogenase
