# Supplementary material for: Dietary management and major clinical events in patients with longchain fatty acid oxidation disorders enrolled in a phase 2 triheptanoin study
Source: Clin Nutr ESPEN. Author manuscript; Available in PMC 2021 Nov 4. (PMC8567087; doi:10.1016/j.clnesp.2020.11.018)
Supplement: Supplement 1 [file NIHMS1728431-supplement-Supplement_1.docx]

Supplementary appendix

Physician questionnaire

| UX007-CL201 patient information | | |
| --- | --- | --- |
|  | Patient identification number form UX007-CL201 |  |
|  | Date of enrollment into UX007-CL201:  Please use format: DDMMMYYYY (i.e., 05JAN2018) |  |
|  | Patient summary: age, disease, presentation, topline summary of clinical status leading to their enrollment in this study (~50 words): | |

| Referring investigator information | | | | | |
| --- | --- | --- | --- | --- | --- |
| Investigator title: | Investigator first name: | | Investigator last name: |  |  |
| Institution/practice name: | | | Department: |  |  |
| Address (line 1): | | | | |  |
| Address (line 2): | | | | |  |
| Town/city: | | State/county/province: | | |  |
| ZIP/postcode: | | Country: | | |  |
| Phone: | | Fax: | | |  |
| Email: | | | | |  |
| Medical specialty of the referring physician | | - Neurologist/neuromuscular specialist - Pediatrician - Geneticist - General practitioner - Other – please specify: | | |  |
| Practice setting of the referring physician: | | - Hospital (university) - Hospital (community) - Private clinic - SMO - Specialist center (please specify): - Other (please specify): | | |  |

| The following questions all refer to the 18 months preceding the patient’s initiation of UX007 (triheptanoin) as part of the CL201 study | | | |
| --- | --- | --- | --- |
|  | Physician primarily responsible for management of the patient’s metabolic condition: |  |  |
|  | Was the patient managed by a trained metabolic physician? | - Yes - No, specify: |  |
|  | Was the patient’s diet managed by a trained metabolic dietician? | - Yes - No, specify: |  |
|  | How often did the patient/caregiver interact with the dietician within the 18 months prior to enrollment to UX007-CL201? | - 0 interactions - 1–3 interactions - 4–6 interactions - 7–9 interactions - ≥10 interactions |  |
|  | Was the patient taking MCT? | - Yes - No |  |
|  | For patients taking MCT, what was the range of % DCI from MCT in the 19 months prior to enrollment? |  |  |
|  | For patients not taking MCT, please provide a reason the patient was not on MCT? Please check all that apply | - Lack of tolerability - Lack of efficacy - Patient choice - Physician choice - Other, specify: |  |
|  | Was the patient’s dietary management reasonably optimized? | - Yes - No, specify: |  |
|  | Did the patient generally comply with the dietary recommended dietary management? | - Yes - No, specify: |  |

| Dietary analysis information | | | | | | | |
| --- | --- | --- | --- | --- | --- | --- | --- |
| For the 18 months prior to enrollment into the CL201 program, please provide any dietary analysis that was completed | | | | | | |  |
| Date of visit DDMMMYYYY | Total caloric Intake (kcal/kg/day) | Fat from MCT  g/kg/day (%) | Other fat g/kg/day (%) | Total fat g/kg/day (%) | Carbohydrate g/kg/day (%) | Protein g/kg/day (%) |  |
|  |  |  |  |  |  |  |  |
|  |  |  |  |  |  |  |  |
|  |  |  |  |  |  |  |  |
|  |  |  |  |  |  |  |  |
|  |  |  |  |  |  |  |  |
|  |  |  |  |  |  |  |  |

| Overall dietary management and assessment | |
| --- | --- |
| In your professional opinion, was the patient medically optimized via diet in the 18 months prior to enrollment?   - Yes - No, specify why not: |  |
| Do you believe the improvement your patient experienced while enrolled in the UX007-CL201 phase 2 study in either the reduction in major clinical events or duration, improved exercise tolerance, and/or quality of life was due to UX007 treatment at a target of 30% of calories?   - Yes - No   Please explain your opinion:  If no, was the improvement due to one of the following (check all that apply):   - Change in diet - Other incidental management - Other, explain: |  |

| Additional comments | |
| --- | --- |
| Please feel free to make any other comments below: |  |

| Questionnaire completion | | |
| --- | --- | --- |
| Investigator name: | Site name: |  |
| Completed by (print name) | Title: |  |
| Date completed: | Telephone & email: |  |
| Signature: | |  |

Supplementary table 1

Patient demographics^a^

| Characteristic | n (%) |
| --- | --- |
| Age group, years |  |
| 0–1 | 2 (6.9) |
| >1–6 | 13 (44.8) |
| >6–18 | 8 (27.6) |
| >18 | 6 (20.7) |
| Gender |  |
| Male | 17 (59) |
| Female | 12 (41) |
| LC-FAOD subtype |  |
| VLCAD | 12 (41) |
| LCHAD | 10 (35) |
| CPT-II | 4 (14) |
| TFP | 3 (10) |
| Prior treatment with MCT |  |
| Yes | 27 (93) |
| No | 2 (7) |
| Clinical manifestations |  |
| Skeletal myopathy | 25 (86) |
| Hepatic disease | 3 (10) |
| Cardiac disease | 2 (7) |
| Disease history^b^ |  |
| Rhabdomyolysis | 26 (90) |
| Muscle pain | 22 (76) |
| Exercise intolerance | 21 (72) |
| Hypoglycemia | 18 (62) |
| Muscle weakness | 16 (55) |
| Cardiomyopathy | 13 (45) |

^a^ N = 29.

^b^ Occurred in ≥10 patients.

CPT-II = carnitine palmitoyl transferase 2; LC-FAOD = long-chain fatty acid oxidation disorders; LCHAD = long-chain 3 hydroxyacyl-CoA dehydrogenase; MCT = medium-chain triglycerides; TFP = trifunctional protein; VLCAD = very long-chain acyl-CoA dehydrogenase.

Supplementary table 2

Annualized MCE event rate and event-day rate

| **Statistics** | **Pre-triheptanoin** | **Triheptanoin** | **p-value** | **% change** |
| --- | --- | --- | --- | --- |
| Annualized event rate, events/year |  |  |  |  |
| Mean | 1.690 | 0.877 | 0.0208 | −48.1 |
| Median | 1.333 | 0.659 | – | −50.6 |
| Q1, Q3 | 0.667, 2.225 | 0.000, 1.311 | – | – |
| Annualized event-day rate, days/year |  |  |  |  |
| Mean | 5.961 | 2.964 | 0.0284 | −50.3 |
| Median | 5.332 | 1.244 | – | −76.7 |
| Q1, Q3 | 0.667, 8.665 | 0.000, 4.666 | – | – |

The annualized event rate (events/year) and annualized event-day rate (days/year) were calculated for the 78 weeks before triheptanoin initiation and during triheptanoin treatment. If subject's age is <18 months at UX007 initiation, the pre-triheptanoin period will be between the birthdate and the date before UX007 initiation date. p-values were calculated using a two-sided paired t-test comparing the pre-triheptanoin and triheptanoin periods.

^a^ N = 29

MCE = major clinical event; Q = quartile.
