# Supplementary material for: Dietary management and major clinical events in patients with longchain fatty acid oxidation disorders enrolled in a phase 2 triheptanoin study
Source: Clin Nutr ESPEN. Author manuscript; Available in PMC 2021 Nov 4. (PMC8567087; doi:10.1016/j.clnesp.2020.11.018)
Supplement: Supplement 2 [file NIHMS1728431-supplement-Supplement_2.docx]

**Supplementary data**

**UX007-CL201 patient flow diagram**
